# Information Technology Governance Domains in Hospitals: A Case Study in Iran

**DOI:** 10.5539/gjhs.v7n3p200

**Published:** 2014-11-30

**Authors:** Mehraban Shahi, Farahnaz Sadoughi, Maryam Ahmadi

**Affiliations:** 1 Department of Health Information Management, School of Health Management and Information Sciences, Iran University of Medical Sciences, Tehran, Iran; 2 Department of Health Information Management, Hormozgan University of Medical Sciences, Bandarabbas, Iran; 3 Health Management and Economics Research Center, School of Health Management and Information Sciences, Iran University of Medical Sciences, Tehran, Iran

**Keywords:** decision making, hospital, information technology, information technology governance

## Abstract

IT governance is a set of organizational structures ensuring decision-making rights and responsibilities with regard to the organization’s IT assets. This qualitative study was carried out to identify the IT governance domains in teaching hospitals affiliated to Iran University of Medical Sciences. There were 10 heads of IT departments and 10 hospital directors. Semi structured interviews used for data collection. To analyze the data content analysis was applied. All the interviewees (100%) believed that decisions upon hospital software needs could be made in a decentralized fashion by the IT department of the university. Most of the interviewees (90%) believed that there were policies for logistics and maintenance of networks, purchase and maintenance, standards and general policies in the direction of the policies of the ministry of health and medical education. About 80% of the interviewees believed that the current emphasis of the hospital’s IT unit and the hospital management for outsourcing of services were in the format of specialized contracts and under supervision of the university Statistic and IT department. A hospital strategic committee is an official organizational group consisting of hospital executives, heads of IT and multiple functional areas and business units in a hospital. In this committee, “the head of hospital” acts as the director of IT activities and ensures that IT strategies are alignment with the hospital business strategies.

## 1. Introduction

Nyamboga and Kemparaju (2002) defined information technology (IT) as a collective term for different technologies involved in processing and transmitting information including computing, telecommunicating and microelectronics (Nyamboga & Kemparaju, 2002). In this regard, health information technology addresses the broad use of health IT within the health care industry to improve the quality of health care, prevent medical errors, reduce health care costs, increase administrative efficiencies, decrease paperwork, and expand access to affordable health care. To attain the afore-mentioned objectives in health organizations, one of the topics discussed broadly in the literature is IT governance (AbuKhousa & Al-Qirim 2012; Bodnar, 2003; Cater-Steel & Global, 2009). IT governance is a set of organizational structures ensuring decision-making rights and responsibilities with regard to the organization’s IT assets (Weil & Ross, 2004; Wilkin & Chenhall, 2010). In 2006, a global survey of 695 organizations was conducted by the IT Governance Institute. According to the survey, 87 percent of the organizations believed that the information technology is required to be expressed in the vision and business strategy of organizations (Abu-Musa, 2006; ITGI, 2006). IT governance helps to ensure that the information technology supports business goals, optimal investments and proper management of IT-related risks and opportunities (Kumbakara, 2008; Van Grembergen & De Haes 2009). Neumann et al. (1999) shows that the role of IT is very important for organizations. The IT has potential to provide services and is a key driving force for business strategy and performance capabilities (Fletcher, 2006). Health care organizations benefiting from this technology and the government strategy ensure that investments in technology will lead to added value for organizations (Neumann et al., 1999; Rusu & Tenga, 2010). Johnson and colleagues (2002), to emphasize the above mentioned points, state that many senior managers of organizations rely on IT based initiatives to control health care systems such as medical errors, cost increase, unstable quality, inefficiency, clinical job satisfaction guarantee and increased defects. Health Information Management Systems Society believes that through the transition of health care systems to an appropriate use of information technology the longevity increases, health care outcomes improve and costs will be reduced (Cater-Steel & Global, 2009; HIMSS, 2008). Health care systems are facing significant challenges associated with information systems (Asadi & Mastaneh, 2012). Most sections of hospital information systems lack proper management, making the conditions for development of hospital information systems unsuitable. Additionally, information systems in health sector face problems such as poor management of the project, imbalanced allocation of IT budgets, poor operational management of IT, and security management and data protection. So, IT governance frameworks can provide proper solution for many of such challenges (DeLone & McLean, 1992; Kuhn et al., 2007; Lapão, 2007). Five IT governance domains are examined, including IT Strategic Alignment, IT Value Delivery, IT Resource Management, IT Risk Management, and IT Performance Management (Bradley et al., 2012; Fletcher, 2006). Monitoring and assurance methods guarantee the executive management and the directing board for acquiring enough information concerning the IT performance of the hospital on time. IT organizational structure and human resources management ensure that they support the hospital’s IT resource management. Monitoring and assurance practices ensure that the directing board and executive management receive sufficient and timely information about IT performance management. IT resource investment, use, and allocation ensure alignment with the hospital’s IT value delivery. Risk management practices ensure that the hospital’s IT related risks are managed in a proper manner. According to the above, the present study investigated domains of IT governance in teaching hospitals affiliated to Iran University of Medical Sciences.

**Figure 1:**
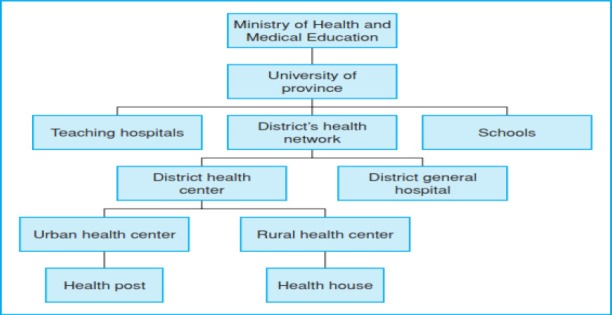
Organizational chart: Ministry of Health and Medical Education of the Islamic Republic of Iran (Mehrdad, 2009)

**Figure 2:**
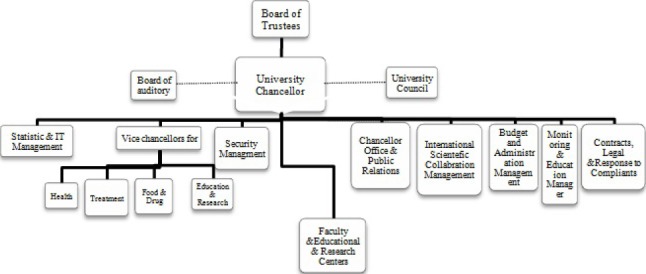
Organizational chart: Iran University of Medical Sciences(IUMS 2014)

**Figure 3:**
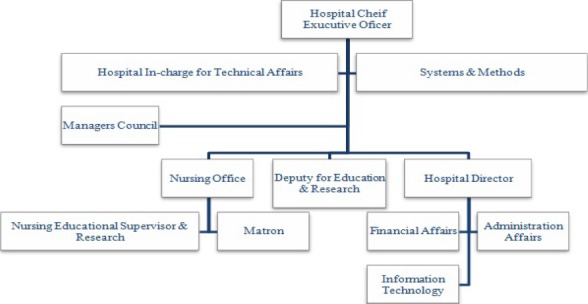
Organizational chart: teaching hospitals in Iran (HKC, 2014)

Figure 1 depicts the organizational structure of the Iranian Ministry of Health and Medical Education (MOHME).The organizational chart of medical universities – in Iran - is shown in Figure 2; and finally, Figures 3 shows the structure related to the teaching hospitals in the universities supervised by the ministry.

The structure of MOHME shows that the medical universities are supervised by MOHME (Figure 1). The structure also depicts that teaching hospitals are managed under the supervision of the medical universities (Mehrdad, 2009). So, it is the same for the hospitals (teaching and non-teaching) affiliated to Iran University of Medical Sciences.

In MOHME, the Office of Statistic and Information Technology (OSIT) makes policies, controls and supports IT related affairs at the level of medical universities as well as hospitals affiliated to the medical universities. In each of the medical universities, there is a bureau called Statistic and Information Technology (SIT) which customizes the MOHME IT policies according to the needs of the university and supervises IT units in different sections of the university. SIT is directly managed by the president of the university. In teaching and non-teaching hospitals IT unit is directly managed by the CEO of the hospital.

## 2. Methods

This qualitative study was conducted among 10 heads of IT departments and 10 hospital directors affiliated to Iran University of Medical Sciences (IUMS). The required data was collected using face to face semi structured interviews. The interview questions consisted of two parts: the first part was related to the demographic information and the second part pertained to main questions related to the study objectives. The interview tool was developed after investigation of reliable resources including literature, guidelines, reports retrieved from the internet by the research team. The objective of this effort was to identify the IT governance domains in the hospitals. The tool was validated by three experts. During the interview, participants were assured that all information will remain confidential. Content analysis was applied in order to analyze the data. First, themes and subthemes were extracted from the interviews and then the main themes related to the study objectives were identified.

## 3. Results

Demographic frequencies showed in Table 1. Results related to the determination of domains of IT governance in hospitals are shown in Table 2.

**Table 1:** Interviewees: demographic information

Interviewee Position	Degree			Field of Study						

	BSc.	MSc.	Doc	Health Services Management	Nursing	Computer-Software	Electronic Engineering	Medical Doctor	Computer-Hardware	Medical Informatics
**HD**	3	3	4	4	2	-	-	4	-	-
**ITM**	9	-	1	-	-	5	1	1	3	-
**DSIM**	-	-	1	-	-	-	-	-	-	1

**Total**	12	3	6	4	2	5	1	5	3	1

**HD**: Hospital Director, **ITM**: IT Manager, **DSIT**: Director of Department of Statistics and Information Technology

**Table 2:** Themes and subthemes derived from the interviews

**Themes**	**Subthemes**
Decision making in the area of Information Technology	Top down decision making
	Bottom up decision making
Decision makers in the area of IT infrastructure	-
Decision makers in the field of software requirements in hospitals	Intersectional decision makers
	Intersectional decision makers
Decision makers in the field of information technology investment	-
IT governance structure in hospitals	Informal and taste based structure
	Committee structure
Organizational and managerial structure of IT in hospitals	Organizational chart
	Duty description
The structures of laws, regulations, policies, standards and procedures of IT in hospitals	the existence of developed, documented and clear policy
	Regulating limited and standards-based practices
The process of the development, approval, implementation and maintenance of IT strategy	The development of action plan for hospital
	The development of mission and values of the hospital
	The development of hospital strategic plan for IT department
Methods for monitoring and evaluating the performance of IT in hospitals	Periodic reporting and visiting
	Schedule of performance monitoring
	Satisfaction survey
	Standards
	Field survey
IT contract management in the hospitals	Services outsourcing
	Centralized contract with the University
IT risk management in hospitals	Maintenance management of data and information
	Network Security
	Periodic monitoring
	Physical isolation
	Physical protection

Analysis of the interviews is provided as the themes and subthemes below:

**I. Decision making in the area of Information Technology(IT Resource management domain and IT strategic alignment domain)**

About 80% of the interviewees emphasized on the importance of decision making in the field of IT and as well as on how to make decisions in the present and future. They believed that these decisions can be made by senior managers in a top down fashion or by expert committees in a bottom up one and then the hospital operational managers are to execute the decisions. In this regard an interviewee stated:

“We have an IT committee which its members include chief and manager of the hospital, teaching assistant, a medical representative and a representative of IT. In addition, according to the subject, the opinions of experts from either inside or outside of the hospital may be used for decision making in especial cases.”

Additionally, more than 60% of the interviewees mentioned the necessity of need assessment prior to decision making by any of the above mentioned committees. One interviewee, for instance, stated:

“Prior to any action, needs announced by different units should be collected either manually or through an automated system; and to be provided to the committee. It is the only way that the outcome of the committee’s decisions can help senior managers to make the right decision.”

**II. Decision makers in the area of IT infrastructure(IT Resource management domain and IT strategic alignment domain)**

Almost all interviewees (90 percent) believed that main and final decisions in the area of IT infrastructure - including network-related issues and domestic IT services - are to be made by the heads of the IT department because the issue to be decided upon is a special and technical one. In this regard one expert stated:

“The issue of infrastructure is a specialized domain of the IT department; however, sometimes recommendations from experts outside of the hospital are needed.”

It is worth noting that hospitals are allowed to select and make decision on the field of network and hardware only within the overall policy framework determined by the university. In this regard, one expert stated:

“We have no limitation regarding the selection of the software for hospital information system or for management information system. But there are frameworks chosen by the hospitals that should be considered in terms of hardware and compliance with other information systems.”

**III. Decision makers in the field of software requirements in hospitals(IT Resource management domain and IT strategic alignment domain)**

Almost 100% of the interviewees believed that decisions in the field of software requirements for all hospitals can be made by the IT department in the university in either an intersectional centralized way. This process is solely related to software used uniformly in all hospitals such as HIS software. But in the case of non uniformed software used specifically for one hospital, decisions may be made intersectionally by hospital, in adherence to upstream policies and regulations.

In regard with decision making on the intersectional preparation for software, one interviewee suggested:

“A positive point in our hospital is that we have two physicians in the IT department who, along with the engineers, develop hospital information system. This makes the program so practical and useful for medical needs.”

**IV. Decision makers in the field of information technology investment(IT value delivery domain)**

About 40% of the interviewees considered the development of optical fiber, new servers and similar cases as the examples of the investment in the field of information technology. They believed that the decision making in such areas is of the senior managers of the organization and the IT department is required to provide necessary expert and professional explanation to justify related officials. In this regard, one interviewee stated:

“In the past year, we were allocated annual certain amounts of money for planning to develop the system. And we used to develop action plans to determine what to be put on the agenda.”

Contrary to the above statement, some interviewees (20 percent) believed that the hospital considers the development of IT as a cost but not investment which is due to insufficient awareness of managers about the IT field.

In regard with macro and integrated investments, one expert stated:

“In order to invest in new initiatives in the field of integrated technologies, the department in charge for suggesting the initiatives for the whole the university is the department of statistics and Information Management and the university’s board of directors and board of trustees are the final decision makers.”

**V. IT governance structure in hospitals(IT resource management domain and IT strategic domain)**

Some interviewees (30 percent) believed that the IT governance has an informal and taste-based structure varying from one hospital to another. In this regard, therefore, the hospital’s IT managers in collaboration with hospital director determine domestic regulations of the hospital based on the particular circumstances of the hospital. However, some other interviewees believed that the current governance structure is the IT committee within the hospitals.

**VI. Organizational and managerial structure of IT in hospitals(IT resource management domain and IT strategic alignment)**

About 80% of the interviewees believed that the job description of the staff working in the IT department of the hospital was specified and predetermined. However, some others(20 percent) mentioned that there were not sufficient number of staff covering the duties and sometimes one had to do several tasks. Meanwhile, some other interviewees believed that there were not specified job descriptions for IT staff in their hospitals.

In regard with the organizational chart and the determination of the authority limits and responsibilities of senior IT staff and the subordinates, the interviewees(70 percent) believed that there was no approved organizational chart for all hospitals; however, they (100 percent)believed that IT unit was supervised by the management of the hospital.

**VII. The structures of laws, regulations, policies, standards and procedures of IT in hospitals(IT strategic alignment domain)**

Almost all interviewees (90 percent) believed that there were policies for purchase, support and maintenance of the network as well as standards and general policies alignment with the policies of the Ministry of Health and Medical Education.

In this regard, one interviewee stated:

“Our overall policy in this area has been set based on three objectives. First, the convenience of customers, second, the convenience of users and third, reduction of costs…”

Meanwhile, a few number of the interviewees (20 percent) pointed out that there was no documented general policy for the IT department in their hospitals. Instead, they had tried to use a set of standards as a basis for their practices and also developed some procedures for their convenience.

**VIII. The process of development, approval, implementation and maintenance of IT strategy(IT strategic alignment domain)**

Some interviewees (40 percent) believed that there was no specified strategic plan for the IT department in their hospitals. In this regard, one interviewee stated:

“… Although the plans we have in most cases are unwritten/undocumented, in the IT department there are verbal agreements according which the system is managed and maintained. It is not a strategic plan….”

Some believed that such situation was also true regarding the action plan. As one expert stated:

“…The action plan has not been written formally and always we plan hastily because of an external pressure… “

However, some other interviewees believed that the IT departments in their hospitals had developed strategic plans. For instance:

“…a strategic plan has been developed within the framework of the clinical governance book through which we try to achieve our goals.”

In addition, another participant stated:

“A set of documents relating to data accreditation and plans to be fulfilled by IT department until the next year was formulated in shape of a plan. But not completely fulfilled yet. … “

Additionally, in regard with the development of the action plan, some interviewees (20 percent) believed that the action plan had been developed based on the hospital strategic plan. In this regard, one interviewee stated:

“We have started to develop the action plan since one year ago but before that time we had neither action plan nor strategic plan…but now we are moving forward successfully according to the plan. “

Some interviewees (40 percent) pointed to the development of mission and vision statements, values and objectives of the hospital. In this regard one expert stated:

“Providing quality care in a short time at the lowest price is the mission statement of the hospital.”

**IX. Methods for monitoring and evaluating the performance of IT in hospitals(IT performance management domain)**

Some experts (50 percent) mentioned that monitoring and evaluation of their hospitals was conducted within periodic inspections and the related results were reported to the hospital management. In this regard, one of the experts stated:

“Annual reports and activities conducted by the department of IT are provided to the hospital manager in the form of periodic reports and he informs the chief of the hospital accordingly.”

In this regard, other interviewee stated that specified forms were developed to review the timing of performance and activities of the IT department in order to control the time of each activity and report to the manager.

Finally, another participant considered the annual users satisfaction survey as a tool for monitoring and evaluating the performance of the IT department in the hospital and stated:

“The users who most frequently use the system, such as the staff working in the department of financial affairs, medical records, admission, inpatient wards and Para- clinic units, are randomly selected and are given the satisfaction survey form annually. The analysis of their criticisms and suggestions help us for further planning.”

Another interviewee considered the standards as an appropriate indicator for monitoring and evaluating the performance of the IT department in the hospital and finally one interviewee stated field surveys as a method for monitoring and evaluating the hospital IT.

**X. IT contract management in the hospitals(IT value delivery domain)**

About 80% of the interviewees believed that currently the focus of hospitals’ IT departments and hospital directors was on the services outsourcing within the framework of specialized contracts under the supervision of the university IT department and there is no other type of form. In this regard, one interviewee stated:

“Service Level Agreements for outsourcing are generally determined and along with an attached checklist form to the agreement draft are sent from the headquarters.”

Some other interviewees (20 percent) state decentralized contracts between companies providing IT services and the Medical Sciences University. In such cases, the hospital does not supervise the contract. But in the case of the outsourcing contracts between the hospital and a specific company, some mentioned that the university supervised the process as the inspector of the contract. In regard with the items included in the contracts, one expert stated:

“…The Service Level Agreement is considered in the contracts and it is exactly mentioned that which programs should be covered…we also control them according to our checklist and express our level of satisfaction of the service provided by the contractor.”

**XI. IT risk management in hospitals(IT risk management domain)**

One interviewee believed that currently the issue of avoiding missing information and data was the only subject considered as the risk management for IT in the hospital.

In this regard, it has been said:

“…missing may be occurred regarding data, service or resource and we have tried to have plan for all of them.”

In regard with avoiding missing data, another interviewee added:

“…every week we get back up of the server and save it on a DVD.”

Another significant point mentioned by the interviewees regarding the risk management was the issue of the network security. In this regard, one interviewee stated:

“…we use antivirus for network security. We signed a contract with a company, bought original antivirus, then we installed it and check it every once in awhile.”

Periodic monitoring was another issue addressed in the field of risk management. As an example:

“…systems connected to the internal network are periodically monitored to see if USB is not put on them.If this violation occurs, we deal with them”

The physical isolation of the server was another issue emphasized by the interviewees. Finally, the interviewees pointed to physical protection such as the existence of firefighting and fire alarm and heat detector systems.

## 4. Conclusion

The identification of the role and insight of IT stakeholders and senior managers of hospitals in major IT decisions is an important issue in health care organizations. To create such involvement and communication, the roles and responsibilities of the key actors should be clearly defined through decision-making structures. Decision making structures clearly show responsibilities and accountability consistent with accepted organizational structures of the IT governance. One of such structures is the hospital-level IT strategic committee. The strategic committee is an official organized group of hospital executives, managers of IT and multiple functional areas and business units in the hospital. In this committee, “the head of the hospital” acts as director of IT activities and ensures that the IT strategy is associated with the hospital business strategy. This conclusion is supported by the fact that hospitals have clearly identified Information Technology Governance domains factors as possible threats to the success of an IT governance framework. Processes in hospitals have been done repeatedly following regular pattern but not well documented and communicated throughout the hospitals. The researchers suggest the IT unit at teaching hospitals affiliated to IUMS to formulate a framework for IT governance in cooperation with SIT of the university and OSIT.
